# Raising Public Awareness: The Role of the Household Sector in Mitigating Climate Change

**DOI:** 10.3390/ijerph121013162

**Published:** 2015-10-20

**Authors:** Shis-Ping Lin

**Affiliations:** Public Affairs Section, National University of Kaohsiung, Kaohsiung 811, Taiwan; E-Mail: splin@nuk.edu.tw; Tel.: +886-7-5919555; Fax: +886-7-5919554

**Keywords:** global warming, climate change, theory of planned behavior, household, mitigate

## Abstract

In addition to greenhouse gas emissions from the industrial, transportation and commercial sectors, emissions from the household sector also contribute to global warming. By examining residents of Taiwan (N = 236), this study aims to reveal the factors that influence households’ intention to purchase energy-efficient appliances. The assessment in this study is based on the theory of planned behavior (TPB), and perceived benefit or cost (BOC) is introduced as an independent variable in the proposed efficiency action toward climate change (ECC) model. According to structural equation modeling, most of the indicators presented a good fit to the corresponding ECC model constructs. The analysis indicated that BOC is a good complementary variable to the TPB, as the ECC model explained 61.9% of the variation in intention to purchase energy-efficient appliances, which was higher than that explained by the TPB (58.4%). This result indicates that the ECC model is superior to the TPB. Thus, the strategy of promoting energy-efficient appliances in the household sector should emphasize global warming and include the concept of BOC.

## 1. Introduction

Greenhouse gases (GHGs) can be naturally released (e.g., through volcanic activity) or stored (e.g., in peat). Anthropogenic GHG emissions have increased since the pre-industrial era, driven largely by economic and population growth [1], and it is difficult for nature to balance the increased concentration of GHGs caused by these activities [2]. Notably, Dietz *et al.* [3] emphasized that the household sector is one of the major emitters of GHGs; direct energy use by households is responsible for approximately 38% of the overall CO_2_ emissions in the U.S., which amounted to approximately 626 million metric tons of carbon in 2005. Household energy use in the U.S. alone thus accounts for approximately 8% of total global emissions and surpasses the total emissions of any other country, except China. By contrast, in OECD countries, households are responsible for 20% of total CO_2_ emissions [4]. Many governments have thus set policies that aim to reduce household energy use and GHG emissions. Despite these policymakers’ efforts, household energy consumption continues to increase, and more effective energy strategies seem to be warranted to reduce household GHG emissions [5].

Mitigating global climate change would require substantial and sustained reductions in GHG emissions [1]. The majority of mitigation or adaptation strategies for climate change are directed at long-term options, such as introducing new low-carbon technologies or creating cap-and-trade regimes for emissions. Most people believe that climate change and sustainability are important problems, but too few global citizens engaged in high-GHG-emitting behaviors to halt the increasing flow of GHGs and other environmental problems [6]. O’Riordan [7] has argued that certain carbon-reducing behaviors are not easy to carry out; thus, humans seem to have strong intentions to avoid immediate dangers but weak intentions to avoid long-term threats, a phenomenon that Lin [8] has called the gap between global issues and personal behaviors.

Household energy use affects the environment primarily through the burning of fossil fuels either directly or in the generation of electricity. The decisions to reduce households’ energy consumption by choosing energy-efficient electric appliances could contribute to reducing environmental impacts [9]. Thus, this study aimed to determine which factors influence households’ intentions to purchase energy-efficient appliances and to offer some suggestions for how the household and industrial sectors might reduce energy consumption and GHG emissions.

## 2. Literature Review

### 2.1. The Theory of Planned Behavior and Modified Models

The theory of planned behavior (TPB) is an extension of the theory of reasoned action [10,11] and is meant to help explain and predict people’s intentions and behaviors [12]. According to the TPB, attitudes toward behavior, subjective norms (SN), and perceived behavioral control (PBC) are usually found to predict behavioral intentions; moreover, intentions in combination with PBC are then used to explain variance in behavior [13]. 

A central factor in the TPB is the individual’s intention to engage in a given behavior. Intentions are assumed to capture the motivational factors that influence behavior; they are indications of how hard people are willing to try, of how much of an effort they are planning to exert, in order to perform the behavior. Generally, the stronger the intention to engage in a behavior, the more likely the occurrence of the behavior will be. However, a behavioral intention will be expressed as a behavior only if the behavior in question is under volitional control—that is, if the person can decide at will whether to perform the behavior [14].

The TPB has often been used to examine the pro-environmental intentions or behaviors of various people [8]. Using the TPB and the norm activation model (NAM; [15]) to examine the energy-saving behavior of households, Abrahamse *et al.* [16] concluded that households use energy in not only direct (e.g., gas, electricity, and fuel) but also indirect (e.g., consuming and disposing of goods) ways and found that the variables of both the TPB and the NAM can explain energy-saving intentions.

Klöckner *et al.* [17] studied energy consumption and transportation and concluded that the TPB effectively explained the variation in student passengers’ travel mode of choice. Tikir *et al.* [18] found that the independent variables of the TPB also explained the intention to use public transport. Kerr *et al.* [19] indicated that the behavioral intention of the TPB was the strongest predictor of private vehicle use.

In addition to energy use, the TPB has been applied in some studies to other pro-environmental intentions and behaviors. Aertsens *et al.* [20] reviewed studies on pro-environmental behaviors based on the TPB and the modified TPB models that examined personal determinants of organic food consumption. Aertsens *et al.* [20] concluded that organic food purchases were positively and significantly related to the intention to purchase such food. 

In studies of water conservation behaviors, the TPB successfully predicted intentions to save water, and the variables of the TPB were positively and significantly correlated with water conservation intentions [21,22,23]. However, the TPB was less successful in predicting the intention to install water-efficient appliances [21].

A meta-analysis from Armitage *et al.* [24] supported the efficacy of the TPB as a predictor of intentions and behavior and suggested that additional normative variables may increase the predictive power of the normative component of the model. Nevertheless, Bamberg and Möser [25] posited that bias may have led to an overestimation of the intention–behavior correlation.

The TPB has been applied to a wide range of human behaviors [26], and the TPB and related modified models have often been used to examine the pro-environmental intentions and behaviors of various groups of people [10]. Ajzen [14] noted that the TPB is open to the inclusion of additional predictors if they can be shown to capture a significant proportion of the variance in intentions or behavior after the theory’s current variables have been taken into account. Thus, to advance analysis using the TPB, this study presents variables to include in the TPB model, among others, that might increase its explanatory power.

### 2.2. Constructs of the Research Variables

#### 2.2.1. Environmental Attitude (EA)

Attitude has been defined in a variety of ways, but at the core, it is the notion of evaluation. Thus, attitudes are commonly viewed as summary evaluations of objects along a dimension ranging from positive to negative [27]. As a general rule, more favorable attitudes toward a behavior should produce stronger individual intentions to perform the behavior [28]. Thus, general behavior should be more closely related to a general environmental attitude (EA) [17]. Ha *et al.* [29] indicated that the attitude toward an energy-efficient product has a stronger effect on intentions than the subjective norm component. To determine the predictors of purchasing energy-efficient appliances, both the specific EA under the circumstances of global warming and the attitude of the individual toward purchasing energy-efficient appliances were identified in the efficiency action toward climate change (ECC) model.

#### 2.2.2. Subjective Norm (SN)

SN is defined as perceived social pressure and is based on an individuals’ perception of whether other important people in their life would want them to perform a behavior. SN have significantly predicted intentions in research on weight loss [30], dishonest actions of college students [31], adolescent bicycle use for transportation [32], consumer attitudes [33], and the effect of environmental knowledge [34]. However, Whitmarsh *et al.* [35] showed that SN cannot significantly predict intentions with respect to certain pro-environmental behaviors. In line with most studies, SN was defined as pressure from the person who will remind the individual to purchase energy-efficient appliances in the ECC model.

#### 2.2.3. Perceived Behavioral Control (PBC)

The role of perceived behavioral control in TPB is the non-volitional elements, which can predict the behavioral intention. PBC comprises two separable components, self-efficacy and controllability, and can be considered a unitary latent variable in a hierarchical factor model [36]. In pro-environmental behavior studies, PBC and other TPB factors accounted for 95% of intentions with respect to conservation behaviors [37]. PBC was also one of the psychological factors that influenced energy-saving behaviors [15]. However, in a study on modes of travel, Tikir *et al.* [18] demonstrated that PBC was not a significant predictor of behavioral intentions. In line with the studies of Abrahamse *et al.* [15] and Lam [22], PBC was defined as the convenience of buying energy-efficient appliances for an individual in the ECC model. 

#### 2.2.4. Perceived Benefit or Cost (BOC)

Ajzen and Driver [38] distinguished behavioral beliefs into two independent types: beliefs about the costs or benefits of engaging in a behavior (instrumental beliefs) and beliefs about positive or negative feelings derived from the behavior (affective beliefs). This study refined the construct of behavioral beliefs by defining instrumental beliefs as the perceived benefit or cost (BOC). Perceived benefits are defined as beliefs about improved conditions or gains produced by a given action, whereas perceived costs involve beliefs about losses engendered by that action.

As with other behaviors, an individual’s BOC influences whether they engage in environmental behaviors; less benefit or more cost reduces the likelihood that individuals will engage in environmental behaviors [39]. For example, consumers who were willing to pay a higher price for emulsion lacquer paints with an environmental label expected a personal advantage from using the labeled products [40]. Similarly, mothers may have strong concerns about environmental and food safety and may thus be willing to pay more for eco-labeled apples [41]. In one study, approximately 49% of Australian tourist respondents were willing to pay extra for hotel accommodations powered by a micro-generation renewable energy supply [42]. In another study, aviation passengers were willing to pay 60 Euro Cent per 100 km when they flew to offset their GHG emissions, on average [43]. Altruistic and environmental attitudes, along with a greater ability to pay, have been shown to reliably predict willingness to pay a premium price for using green electricity [44]. Gaspar *et al.* [45] argued that their results indicated a preference for first considering cost, followed by quality and energy consumption.

This study distinguishes between PBC and BOC and uses different variables to assess them. PBC is concerned with the perceived ability to perform a behavior (or a sequence of behaviors), and it is quite similar to Bandura’s work on self-efficacy [35], whereas BOC indicates that more benefit or less cost would encourage individuals to engage in behaviors based on their behavioral intentions.

For example, although an incandescent light bulb and an LED light bulb emit the same luminous flux, an incandescent light bulb costs about US$1.57 in Taiwan, whereas an LED light bulb costs about US$4.72. The consumer has the perceived ability to buy both types of light bulbs. If the consumers do not have any more information, the cheap product is always purchased. However, including the BOC analysis in the same cases reveals that the annual electricity bill is approximately US$3.14 for an incandescent light bulb and US$1.50 for an LED light bulb. Thus, it is a good deal for the consumer to choose the LED light bulb over the incandescent light bulb if it will be used for more than 2 years.

The ECC model followed the studies of Loureiro *et al.* [41], Dalton *et al.* [42], and Ward *et al.* [46] and identified BOC as follows: energy-efficient appliances were typically more expensive than traditional appliances, but the reductions in energy bills that were achieved by using energy-efficient appliances totaled more than the premium price.

#### 2.2.5. Intention toward Efficiency Actions (IEA)

Pro-environmental behaviors have been grouped into two classes: efficiency behaviors and curtailment behaviors [47]. Efficiency behaviors are one-time investments in efficient infrastructure such as a decision to use energy-efficient light. By contrast, curtailment behaviors are repetitive efforts aimed at reducing resource consumption, such as continually ensuring that lights are turned off in unoccupied rooms. Flemming *et al.* [48] emphasized that efficiency behaviors appear to be of greater benefit than curtailment behaviors, as they tend to result in a greater overall reduction in energy use. This study focuses on efficiency behaviors to determine the factors that influenced the households’ intentions to purchase energy-efficient appliances.

Previous studies have focused on intentions toward efficiency behaviors. For example, Lam [22] used the TPB and modified models to predict people’s intention to save water and found that the TPB alone was insufficient to explain the intention to install a dual-flush controller in toilets, whereas an additional variable, the subjective effectiveness of alternative solutions, was a good predictor for such an intention.

Clark *et al.* [23] argued that all the TPB variables were significantly positively correlated with water conservation intentions and that an additional variable, self-perceived knowledge of climate change, was also significantly related to such intentions. In addition, EAs and concerns regarding future shortages were significant but relatively weak determinants.

Chen *et al.* [49] proposed an integrated model that combined the TPB, the technology acceptance model (TAM), and habit to examine private vehicle users’ switching intentions with regard to public transit and found that the habitual behavior of private vehicle use hinders individuals’ intention to switch from a car or motorcycle to public transit.

Following Lam [22], Clark *et al.* [23], and Chen *et al.* [49], this study considers appliances that Taiwanese citizens continue to use. This study examined intentions to engage in efficiency actions when appliances are replaced, *i.e.*, *in situ*ations in which the individual may be willing to purchase energy-saving appliances.

## 3. Methods 

### 3.1. Research Model

This study was based on the TPB, and oriented from mitigating global warming to develop the model of efficiency action toward climate change (ECC) model. Notably, some studies on environmental behavior have measured intention behavior instead of actual behavior [22,23,49]. This study considered the appliances of households that remained in use and had not been recently replaced. Therefore, this study examines intentions regarding pro-environmental behaviors, not actual behaviors.

Although the TPB allows additional variables to be included in the model, Ajzen [14] clearly outlined the ways in which central model variables should be measured. In the ECC model, all the independent variables of the TPB (EA, SN, and PBC) are presumed to predict the dependent variable (intention toward efficiency actions, IEA). In addition, as an independent variable, BOC is also assumed to predict IEA.

### 3.2. Research Design

The population of Taiwan is approximately 23 million, and the *per capita* CO_2_ emissions are approximately 11.1 t [50], which is close to the OECD average of 12.76 t [51] and far in excess of the global *per capita* emissions of 3.96 t. Direct energy use by Taiwanese households accounted for approximately 27 million metric tons of carbon in 2000 and 32 million metric tons in 2010 [45]. Kaohsiung, the most important industrial metropolis in Taiwan, has an annual average *per capita* CO_2_ emission of 26.3 t [52]. Notably, the majority of CO_2_ emissions in Kaohsiung originate in the industrial sector (68%), followed by the commercial and household sectors (23%). Therefore, the Taiwanese government encouraged households to purchase energy-saving appliances and classified the energy consumption of appliances into five levels. Level one represents the most energy-efficient appliances, which typically have the highest prices. This study aimed to develop the ECC model and applied structural equation modeling (SEM) to confirm the model’s applicability in the study’s sample of Kaohsiung residents.

### 3.3. Research Participants

This study employed a sample of 236 Kaohsiung residents and used questionnaires and face-to-face interviews to collect information. Among the 235 valid responses in this study, females (55.3%) outnumbered males (44.7%). Respondents aged 20–29 years (25.1%), followed by those aged 30–39 years (18.7%). The majority of respondents had completed a college education (36.6%). Of the respondents, 54.9% were not married. The largest group of respondents reported TWD$5,000 (US$175) in personal monthly disposable income. Finally, with respect to religion, the largest category was respondents with “no religious beliefs” (38%), followed by Buddhists (20%) and Taoists (12.6%).

## 4. Results and Discussion

### 4.1. Reliability Analysis

The Cronbach’s alpha for each construct in the ECC model was above 0.7, which complies with the suggestion of Hair *et al.* [53] and indicates acceptable reliability. All constructs showed acceptable consistency: EA (0.927), SN (0.897), PBC (0.920), BOC (0.922), and IEA (0.924).

### 4.2. Confirmatory Factor Analysis

A confirmatory factor analysis was conducted. Hair *et al.* [53] suggested that a critical ratio (C.R.) of 0.7 and above indicates good composite reliability and that an average variance extracted (AVE) value of 0.5 and above indicates a good convergent validity. Doll *et al.* [54] suggested that goodness-of-fit index (GFI) values of 0.8 and above indicate a reasonable fit. Byrne [55] suggested that a comparative fit index (CFI) value of 0.9 and above indicates a good model fit as does a Tucker-Lewis index (TLI) value close to 1 indicates good fit to a model. Byrne [55] also suggested that a root mean square error of approximation (RMSEA) value below 0.08 indicates a comparatively good fit. Joreskog *et al.* [56] suggested that a $x^{2}$/df value below 5 indicates an acceptable model. 

**Figure 1:**
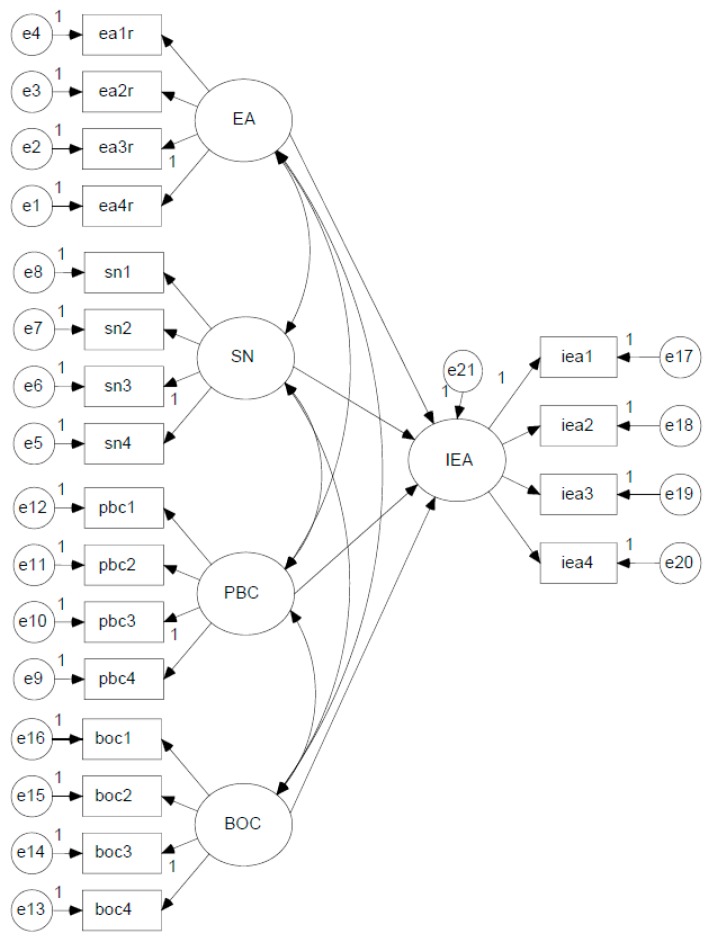
ECC model.

SEM was applied, and most of the indicators in this study fit the corresponding constructs of the ECC model well (shown in Figure 1, Table 1). Squared multiple correlations (SMCs) showed that all the observed variables reflected the constructs (latent variables) effectively (shown in Table 2).

**Table 1:** Confirmatory factor analysis of the ECC model constructs.

Construct	Items	Non-Standardized Factor Loading	Standard Error (SE)	t-Value	C.R.	AVE	$x^{2}$	$x^{2}$/df	GFI	TLI	CFI	RMSEA
Environmental Attitude (EA)	ea1r	1.000			0.930	0.770	13.319	6.659	0.971	0.958	0.986	0.156
	ea2r	1.166	0.069	17.009 ^***^								
	ea3r	1.140	0.070	16.397 ^***^								
	ea4r	1.096	0.075	14.528 ^***^								
Subjective Norm (SN)	sn1	1.000			0.898	0.689	16.694	9.847	0.958	0.910	0.970	0.194
	sn2	1.169	0.094	12.454 ^***^								
	sn3	1.202	0.095	12.716 ^***^								
	sn4	1.168	0.092	12.719 ^***^								
Perceived Benefit or Cost (BOC)	boc1	1.000			0.925	0.757	0.018	0.009	1	1	1	0
	boc2	1.174	0.079	14.806 ^***^								
	boc3	1.206	0.080	15.207 ^***^								
	boc4	1.113	0.081	13.829 ^***^								
Perceived Behavioral Control (PBC)	pbc1	1.000			0.926	0.760	2.791	2.791	0.994	0.986	0.998	0.087
	pbc2	1.333	0.094	14.232 ^***^								
	pbc3	1.343	0.094	14.305 ^***^								
	pbc4	1.352	0.107	12.683 ^***^								
Intention toward Efficiency Actions (IEA)	iea1	1.000			0.930	0.771	3.156	3.156	0.993	0.984	0.997	0.096
	iea2	1.227	0.083	14.789 ^***^								
	iea3	1.207	0.082	14.690 ^***^								
	iea4	1.214	0.091	13.338 ^***^								
Recommended values					> 0.7	> 0.5	the lower the better	<5	>0.8	>0.9	>0.9	<0.08

Note: *** *p* < 0.001.

**Table 2:** The squared multiple correlation (SMC) for each indicator in the construct.

Construct	Items	Average	Standard Deviation	Standardizd Factor Loading	SMC	Subject
Environmental Attitude (EA)	ea1r	4.948	1.755	0.781	0.611	Using energy-saving lamps is not necessary to mitigate global warming *.
	ea2r	5.097	1.677	0.953	0.909	Using an energy-saving refrigerator is not necessary to mitigate global warming *.
	ea3r	4.991	1.694	0.923	0.852	Using an energy-saving washing machine is not necessary to mitigate global warming *.
	ea4r	5.140	1.783	0.843	0.711	Using an energy-saving air conditioner is not necessary to mitigate global warming *.
Subjective Norm (SN)	sn1	4.748	1.692	0.720	0.519	When I buy a lamp, the person whom I concern will remind me to purchase energy saving one.
	sn2	4.914	1.674	0.850	0.723	When I buy a refrigerator, the person whom I concern will remind me to purchase energy saving one.
	sn3	4.970	1.682	0.871	0.758	When I buy a washing machine, the person whom I concern will remind me to purchase energy saving one.
	sn4	5.097	1.633	0.871	0.758	When I buy an air conditioner, the person whom I concern will remind me to purchase energy saving one.
Perceived Benefit or Cost (BOC)	boc1	5.225	1.451	0.744	0.554	An energy-saving lamp is more expensive, but by saving electricity, it is cheaper than using a traditional one.
	boc2	5.383	1.376	0.922	0.849	An energy-saving refrigerator is more expensive, but by saving electricity, it is cheaper than using a traditional one.
	boc3	5.323	1.392	0.936	0.876	An energy-saving washing machine is expensive, but by saving electricity, it is cheaper than using a traditional one
	boc4	5.412	1.388	0.866	0.750	An energy-saving air conditioner is expensive, but by saving electricity, it is cheaper than using a traditional one.
Perceived Behavioral Control (PBC)	pbc1	5.289	1.429	0.733	0.538	It is convenient for me to purchase an energy-saving lamp.
	pbc2	5.157	1.517	0.921	0.848	It is convenient for me to purchase an energy-saving refrigerator.
	pbc3	5.097	1.520	0.926	0.858	It is convenient for me to purchase an energy-saving washing machine.
	pbc4	5.285	1.587	0.893	0.797	It is convenient for me to purchase an energy-saving air conditioner.
Intention toward Efficiency Actions (IEA)	iea1	5.174	1.473	0.748	0.559	When I replace a lamp, I will purchase an energy-saving one.
	iea2	5.293	1.459	0.926	0.857	When I replace a refrigerator, I will purchase an energy-saving one.
	iea3	5.259	1.445	0.919	0.844	When I replace a washing machine, I will purchase an energy-saving one.
	iea4	5.455	1.473	0.907	0.822	When I replace an air conditioner, I will purchase an energy-saving one.

Note: * Reverse questions.

### 4.3. Model Test

#### 4.3.1. Two-Step Appraisal

A two-step appraisal [57] was conducted to test the ECC model. In the first step, a confirmatory factor analysis of the entire model was conducted. Kline [58] argued that a correlation coefficient greater than 0.85 indicates multicollinearity between variables. The correlation coefficients between the latent variables of the ECC model ranged from approximately 0.132 to 0.627 (shown in Table 3), indicating that there were low or medium correlations between the latent variables of the ECC model. In the second step, SEM was conducted to test the ECC model. The values of the fit indices showed good model fit: $x^{2}$[160] = 3.093, GFI = 0.811, CFI = 0.922, TLI = 0.908 and RMSEA = 0.095.

**Table 3:** Standard correlation coefficients between the latent variables of the ECC model.

Latent Variables	Correlation	Latent Variables	Standard Correlation Coefficient
Environmental Attitude	<--->	Subjective Norm	0.196
	<--->	Perceived Benefit or Cost	0.170
	<--->	Perceived Behavioral Control	0.132
	<--->	Intention toward Efficiency Actions	0.296
Subjective Norm	<--->	Perceived Benefit or Cost	0.540
	<--->	Perceived Behavioral Control	0.331
	<--->	Intention toward Efficiency Actions	0.601
Perceived Behavioral Control	<--->	Perceived Benefit or Cost	0.315
	<--->	Intention toward Efficiency Actions	0.627
Perceived Benefit or Cost	<--->	Intention toward Efficiency Actions	0.549

#### 4.3.2. Invariance (Equivalence) Test

To test the stability of the ECC model, the model can be measured across specified groups. For this purpose, an invariance (equivalence) test was used. Vandenberg *et al.* [59] argued that tests for measurement invariance (the associations of observed scores with latent variables) should precede tests of structural invariance (associations of latent variables with one another). This study divided the sample by gender and tested the invariant covariance matrices across gender groups. The results showed acceptable fit ($x^{2}$[387] = 2.383, CFI = 0.882, TLI = 0.884, and RMSEA = 0.077), but the fit was inadequate (CFI and TLI < 0.9). To confirm the equivalence of the ECC model, this study conducted the invariance tests suggested by Vandenberg *et al.* [59]. The results are presented in Table 4.

**Table 4:** Invariance test of the ECC model.

Model	$x^{2}$	df	$x^{2}$/df	CFI	TLI	RMSEA	Δ$x^{2}$	Δdf	ΔCFI	ΔTLI
Configural invariance	723.507	314	2.304	0.910	0.891	0.075	--	--	--	--
Metric invariance	755.145	329	2.295	0.906	0.891	0.075	31.639	15	−0.004	0
Scalar invariance	770.435	349	2.208	0.907	0.899	0.071	15.290	20	0.001	0.008
Invariant uniqueness	772.360	353	2.188	0.907	0.900	0.071	1.925	4	0	0.001
Covariances invariant	787.174	363	2.169	0.906	0.902	0.071	14.814	10	−0.001	0.002
Structural residuals	787.438	364	2.163	0.907	0.902	0.071	0.264	1	0.001	0
Measurement residuals	922.136	387	2.383	0.882	0.884	0.077	134.698	23	−0.025	−0.018

Vandenberg *et al.* [59] claimed that changes in CFI (ΔCFI) between −0.01 and 0.01 indicate that the invariance hypothesis should not be rejected; however, when the differences are between −0.01 and −0.02 or between 0.01 and 0.02, the researcher should be suspicious that differences exist. Little [60] suggested that changes in the TLI (ΔTLI) of −0.05 to 0.05 also indicate that the invariance hypothesis should not be rejected. Nevertheless, Marsh *et al.* [61] and Byrne *et al.* [62] argued that if only minor parts of the tests do not fit the suggested values, the researcher can still postulate the invariance of the model.

The results of the invariance test of the ECC model showed that most of the indicators fit the suggested values from Vandenberg *et al.* [60] and Little [61], except that the value of the ΔCFI of the measurement residual was 0.025 (>0.01). Based on the suggestions of Marsh *et al.* [61] and Byrne *et al.* [62], this study concluded that the ECC model could be measured across gender groups with invariance.

### 4.4. Path Analysis

A path analysis of the ECC model was also conducted. The analysis showed that EA could predict IEA (*p* < 0.01) but that SN, PBC and BOC could more effectively predict IEA (*p* < 0.001). As a result, this study concluded that all the independent variables could significantly influence the dependent variable in the ECC model (results shown in Table 5).

**Table 5:** Path analysis of the ECC model.

	Estimate	S.E.	C.R.	*P*
IEA<-- EA	0.105	0.034	3.056 **	0.002
IEA<-- SN	0.228	0.047	4.908 ***	0.000
IEA<-- PBC	0.333	0.045	7.453 ***	0.000
IEA<-- BOC	0.199	0.053	3.775 ***	0.000

Note: ** *p* < 0.01, *** *p* < 0.001.

### 4.5. Comparison of the TPB and ECC Model

This study conducted SEM to test the TPB variables, and the values of the indicators are as follows: $x^{2}$ = 3.774, GFI = 0.828, CFI = 0.920, TLI = 0.902, RMSEA = 0.109 and parsimony goodness-of-fit index (PGFI) = 0.597. The values of the indicators indicate an acceptable fit to the TPB model.

The ECC model extends the TPB by introducing BOC as an independent variable. Byrne [54] argued that a PGFI value under 0.5 indicates an unacceptable fit to the model. The PGFI value of the ECC model was 0.618, suggesting that the parsimony of the ECC model is acceptable. Moreover, the ECC model explained 61.9% of the variation in IEA, which is greater than that explained by the TPB (58.4%). Thus, the ECC model is superior to the TPB in explaining variation in the intention to purchase energy-efficient appliances. 

## 5. Conclusions and Recommendations

### 5.1. Conclusions

To determine the key factors that might influence individuals’ intention to purchase energy-efficient appliances, this study extended the TPB by introducing BOC as an independent variable to develop the ECC model. All the independent variables of the ECC model (EA, SN, PBC, and BOC) significantly influenced the dependent variable (intention to purchase energy-efficient appliances). According to the invariance test, the ECC model showed stability and measurability across gender groups.

Regarding the comparison between the ECC model and TPB, the model fit indices of both the ECC model and TPB were acceptable, although the ECC model included more variables than the TPB. The parsimony index of the ECC model was also acceptable, and ultimately, the ECC model was superior to the TPB in explaining variation in the intention to purchase energy-efficient appliances.

Although EA was able to predict the intention to purchase energy-efficient appliances, the standardized regression coefficient was only 0.105, and Chin [63] has argued that standardized regression coefficients should be greater than 0.2. This study suggests that energy-efficient appliances should not be promoted merely by making appeals to mitigating global warming or climate change because the effect of EA on the intention of purchasing energy-efficient appliances is insufficient. However, this finding does not indicate that appealing to mitigate global warming or climate change is useless. Rather, promotional strategies should focus on not only global warming but also on concepts such as SN, PBC, and BOC.

The results of this study showed that SN can predict the intention to purchase energy-efficient appliances (*p* < 0.001), as the standardized regression coefficient (0.236) was significant in the ECC model. Thus, the opinions of a person of concern to an individual may influence that individual’s intention to purchase energy-efficient appliances.

The Taiwanese government has encouraged citizens to purchase appliances with “energy-saving” labels. The results demonstrated that PBC can predict the intention to purchase energy-efficient appliances (*p* < 0.001), and standardized regression coefficient (0.333) showed significantly in the ECC model. Thus, popularizing energy-efficient appliances in the electrical market might increase individuals’ intention to purchase energy-efficient appliances.

The results also showed that BOC can predict the intention to purchase energy-efficient appliances (*p* < 0.001) and those energy-efficient appliances are typically more expensive than traditional appliances. However, if an individual perceives the reduction in energy costs from energy-efficient appliances to exceed their premium price, then they may have a higher intention to purchase energy-efficient appliances.

The TPB and modified models had been used to examine various pro-environmental intentions and behaviors. This study revealed that promoting households’ intention to purchase energy-efficient appliances should focus not only on global warming but also on the concepts of subjective norm, perceived behavioral control and perceived benefit or cost. The findings of this study add to the existing literature on pro-environmental behavior by showing the importance of providing appropriate economic incentives in order to encourage households to choose energy-efficient appliances, which would engender energy savings.

### 5.2. Recommendations

Extending previous studies based on the TPB, this study shows that BOC is a good complementary variable for the TPB and that the ECC model is superior to the TPB in explaining the variation in intention to purchase energy-efficient appliances. Thus, to increase households’ intention to purchase energy-efficient appliances and encourage citizens to purchase higher-priced, energy-efficient appliances, the government should help households understand that the energy cost savings of energy-efficient appliances exceed the price premiums of such appliances.

The amount of energy consumed is determined when appliances are brought to a house. One of the critical places within which the household sector can reduce GHG emissions is in the appliance market. This study recommends that the industrial sector provide the energy consumption information of all the appliances and make a comparison list for salespeople and retailers who introduce these appliances to consumers. Households can then compare the energy consumption and cost of the appliances immediately. For example, households could determine how many years it would take for the savings from reduced energy costs to exceed the price premium of an energy-efficient appliance. Such a strategy might increase households’ intention to purchase the most energy-efficient appliances.

### 5.3. Limitations and Further Research

The ECC model is subject to a number of limitations that can serve as starting points for further research. This study focused on the ECC model in the household sector, and future research may extend this model to other fields, such as offices and housing communities. Four types of electrical appliances were used as items in this study, and further research may extend the model to other appliances or equipment.
